# Aptamer‐Based Approaches for Influenza Virus Detection: A Systematic Review

**DOI:** 10.1002/hsr2.72118

**Published:** 2026-03-17

**Authors:** Javad Charostad, Zohreh‐al‐sadat Ghoreshi, Nasir Arefinia, Faranak Salajegheh, Niloofar Farsiu, Mohammad Rezaei Zadeh Rukerd, Mohsen Nakhaie

**Affiliations:** ^1^ Department of Microbiology, Faculty of Medicine Shahid Sadoughi University of Medical Sciences Yazd Iran; ^2^ Student Research Committee Jiroft University of Medical Sciences Jiroft Iran; ^3^ Social Determinants of Health Research Center, Institute for Futures Studies in Health Kerman University of Medical Sciences Kerman Iran; ^4^ Gastroenterology and Hepatology Research Center, Institute of Basic and Clinical Physiology Sciences Kerman University of Medical Sciences Kerman Iran; ^5^ HIV/STI Surveillance Research Center, and WHO Collaborating Center for HIV Surveillance, Institute for Futures Studies in Health Kerman University of Medical Sciences Kerman Iran; ^6^ Research Center of Tropical and Infectious Diseases Kerman University of Medical Sciences Kerman Iran; ^7^ Clinical Research Development Unit, Afzalipour Hospital Kerman University of Medical Sciences Kerman Iran

**Keywords:** aptamer, aptasensors, hemagglutinin, influenza, systematic review

## Abstract

**Background and Aims:**

Influenza remains a globally prevalent respiratory infection that is challenging to diagnose accurately. This systematic review focuses on aptamer‐based tools for influenza virus detection, aiming to overcome the limitations of traditional diagnostic methods.

**Methods:**

Following PRISMA guidelines, we identified relevant studies from official databases (PubMed, Web of Science, Scopus, Embase, Google Scholar) and grey literature sources. The search strategy utilized keywords related to aptamers, systematic evolution of ligands by exponential enrichment (SELEX) techniques, and influenza. The qualitative synthesis included 22 articles out of 1432 studies.

**Results:**

Our analysis revealed diverse aptamer‐based techniques for influenza detection, including fluorescence resonance energy transfer (FRET)‐based aptasensors, electrochemical sensors, and surface‐enhanced raman scattering (SERS)‐based methods. SELEX was the predominant method for aptamer development, ensuring high specificity and adaptability to various influenza strains. Notably, FRET‐based methods demonstrated detection limits as low as 0.43 ng/mL, while electrochemical approaches exhibited high sensitivity with low detection limits in the picomolar range. The findings highlight the advantages of aptamers, including cost‐effectiveness, stability, and rapid synthesis, though challenges related to reproducibility and technological complexity remain.

**Conclusion:**

Aptamer‐based tools offer promising alternatives to traditional diagnostics, especially for rapid and field‐deployable influenza detection. Their adaptability and potential for multiplex detection underscore their role in advancing public health diagnostics. Further research should focus on optimizing sensitivity and simplifying technological requirements for broader clinical applications.

## 1 Introduction

Influenza, a highly contagious respiratory infection, continues to pose significant global health challenges due to its rapid transmission, frequent antigenic variations, and high mutation rates. Seasonal influenza epidemics result in substantial morbidity and mortality, with an estimated 290,000 to 650,000 respiratory deaths annually worldwide [1, 2]. Additionally, sporadic pandemics caused by novel influenza strains, such as the H1N1 outbreak in 2009, further underscore the need for effective diagnostic tools. The ability to rapidly and accurately identify influenza infections is critical for implementing timely treatment, curbing transmission, and managing public health responses. However, existing diagnostic methods face several limitations that hinder their effectiveness [3, 4].

Without reliable, accurate, and quick diagnostic tools with high sensitivity and specificity, it is impossible to stop the negative effects of viral, health, and economic infections. This is also true for effective therapeutic remedies. Accurate results from these diagnostic methods facilitate the implementation of appropriate treatment, quarantine, or a combined approach [5, 6, 7].

The flu season of 2009–2010 led to a pandemic in the 21st century involving the H1N1 virus, commonly referred to as swine flu. This strain originated in swine, serving as the primary host for viral infections [8]. In this context, a notably consequential variant is the avian influenza virus H5N1, causing considerable economic losses and fatalities in domestic poultry. Additionally, under favorable conditions, it can cross the species barrier, leading to a human infection characterized by a substantial mortality rate [9, 10, 11]. These challenges underscore the urgency of developing effective diagnostic tools for early detection, which is essential for timely intervention and management.

The structure of the influenza virus includes two glycoproteins, hemagglutinin (HA) and neuraminidase (NA). HA plays a role in attaching the viral body to the sialic acid on the surface of the target cell, while NA facilitates the desialylation of HA [12]. This process is crucial for completing the infectious cycle by enabling virion discharge and preventing the aggregation of virions caused by HA attachment to sialic acid. Influenza encompasses three distinct strains: A, B, and C. In terms of causing human diseases, influenza type A pandemics are more common compared to influenza B and C, as the latter two strains mainly infect animals [13, 14]. This biological complexity provides the foundation for current diagnostic approaches, as discussed below.

The influenza virus is known for its ability to undergo frequent changes, requiring periodic adjustments to develop an effective vaccine for each year's influenza outbreak. The challenge lies in the variability of the influenza strain. Consequently, early detection becomes the sole crucial factor in addressing this issue [15]. Currently, nucleic acid‐based and antibody tests, such as reverse transcription‐polymerase chain reaction (RT‐PCR) and enzyme‐linked immunosorbent assay (ELISA), are used to diagnose influenza strains [16]. Culture methods are not considered due to the specialized laboratory requirements. The utilized methods also face limitations due to problems such as high cost and the need for advanced equipment [17, 18].

While PCR is a sensitive technique, the chance for cross‐contamination lowers its specificity. ELISA is a more specified technique. However, the time and costs needed for antibody development do not address the issues with multiple antigenic changes in influenza. Moreover, the low stability of antibodies makes them vulnerable to temperature and humidity. Both techniques mentioned also face biosafety issues. All these shortcomings necessitate designing novel diagnostic approaches [19, 20, 21].

Aptamer‐based detection methods have been proposed to address existing issues. This approach has shown high levels of affinity and specificity. Unlike traditional methods, no toxicity has been reported for aptamers. The low cost and time of manufacturing and high stability of the aptamers have made them considerable alternatives for influenza detection [19, 20, 22]. In recent years, numerous aptamer‐based techniques have been developed and used to detect respiratory viral infections. Following the COVID‐19 pandemic, numerous studies have concentrated on developing aptamer‐based technologies for detecting and treating SARS‐CoV‐2‐related infections [23, 24]. A recent study has developed an aptamer‐based technique capable of simultaneously detecting SARS‐CoV‐2, influenza A, and influenza B, offering a prospective view of the potential applications of these methods for multiplex detection strategies [25]. The aptamer consists of multiple nucleic acids or peptide sequences capable of forming strong and specific bonds with particular targets, such as proteins, small molecules, chemicals, and nucleic acids [26]. The purpose of the present study, which was designed and implemented as a systematic review, was to investigate the efficiency of using aptamers in the detection of different influenza virus variants.

## 2 Material and Methods

### 2.1 Eligibility Criteria

**Figure 1:**
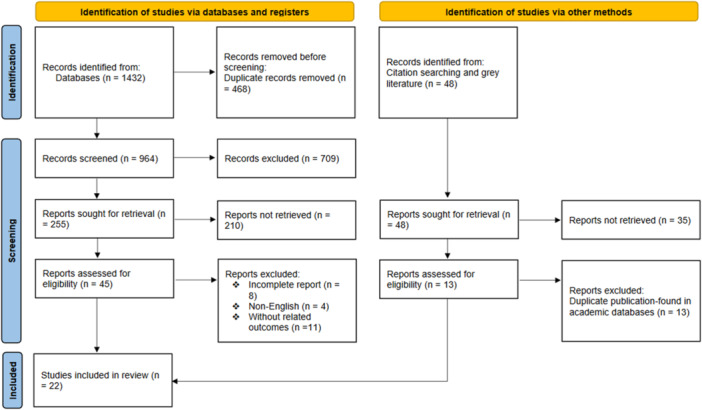
The risk of bias assessment of included studies based on ROBINS‐I tool.

We conducted a systematic review that adhered to the PRISMA guidelines [27]. Any original, peer‐reviewed study investigating the effects of aptamers (including experimental and human studies) in influenza virus detection met the criteria for inclusion. We excluded studies if they were reported as a review study, without a complete paper, or with insufficient data. Figure 1 displays the PRISMA flow diagram, illustrating the process of selecting studies.

### 2.2 Information Sources

In this study, researchers systematically searched official and non‐official literature. For official databases, we searched PubMed, Web of Science, Scopus, Embase, and Google Scholar. Also, in terms of grey literature, we searched preprint studies and some websites like conferencealerts. com, opengrey. eu, and oatd. org. Also, we evaluated the reference lists of the included studies.

### 2.3 Search Strategy

We searched databases and related literature with these MeSH and non‐MeSH keywords: #1 “SELEX Aptamer Technique” or Aptamers or “Aptamer‐based technique”; and #2 flu or influenza.

### 2.4 Selection Process

In the first stage of screening, two researchers (J.C. and Z.G.) evaluated and screened study titles and abstracts. Any disagreement between the two researchers was solved through consultation with a third person. In the next stage, the full text of the studies was evaluated independently by two researchers, and the studies were evaluated based on inclusion criteria. Initial screening was done using EndNote software version 10.

### 2.5 Process of Collecting Data

The data extraction process from the included studies was done independently by three researchers (N.A., F.S., and M.R.Z.R.). The extracted data included the first author's name, the year of publication of the article, the type of aptamer used, the influenza variant, the type of sample examined, and the main results of the study. Any disagreement between the authors involved in this phase was resolved through discussion and consultation.

### 2.6 Assessing the Risk of Bias

The risk of bias in the included studies was evaluated using the Risk Of Bias In Non‐randomized Studies of Interventions (ROBINS‐I) tool [28]. This tool assesses bias across seven domains: confounding, selection of participants, classification of interventions, deviations from intended interventions, missing data, measurement of outcomes, and selection of reported results. Each domain was graded as Low, Moderate, Serious, or Critical risk of bias based on specific criteria outlined in the ROBINS‐I framework. For each study, two independent reviewers (N.A. and F.S.) assessed the risk of bias, and discrepancies were resolved through discussion with a third reviewer (M.R.Z.R.) to ensure consensus.

To evaluate confounding, we considered factors such as sample type variability (e.g., serum, saliva, or buffer) and study design (e.g., in vitro vs. clinical settings) that could influence the reported performance of aptamer‐based detection methods. Selection bias was assessed by examining whether participant or sample selection was representative of the target population, such as influenza‐positive clinical samples. For the classification of interventions, we evaluated the clarity and consistency of aptamer‐based techniques described in each study. Deviations from intended interventions were assessed by checking for adherence to the reported methodology, such as consistent application of SELEX or assay protocols. Missing data were evaluated based on the completeness of reported outcomes, including sensitivity, specificity, and detection limits. Outcome measurement bias was assessed by reviewing the reliability and standardization of detection methods (e.g., electrochemical or fluorescence‐based assays). Finally, the selection of reported results was scrutinized to ensure that all relevant outcomes were reported without selective omission.

**Figure 2:**
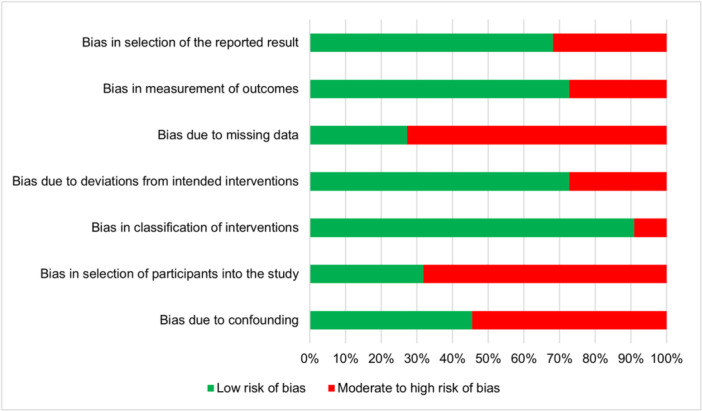
Preferred Reporting Items for Systematic Reviews and Meta‐Analyses flow diagram (2020) of the search process.

The overall risk of bias for each study was determined by the highest risk level assigned to any single domain, as per ROBINS‐I guidelines. A detailed summary of the risk of bias ratings for each of the 22 included studies is provided in Supporting Information Table S1, which includes domain‐specific ratings and an overall risk of bias score for transparency. The results of this assessment are visualized in Figure 2, which presents the risk of bias distribution across the included studies.

### 2.7 Statistical Analysis

Due to the high heterogeneity in the investigated aptamers, the types of samples evaluated, and the results, it was not possible to perform a meta‐analysis, and the study was conducted in the form of a systematic review.

## 3 Results

### 3.1 Study Selection

Database searches yielded a total of 1432 studies. Before the primary screening, 468 articles were eliminated due to duplication. Two researchers initially screened the titles and abstracts of 964 articles, with 709 being excluded based on their incompatibility with the inclusion and exclusion criteria. Two hundred and fifty‐five studies underwent a comprehensive examination of their full texts, and 45 studies were included for the next phase, and finally, 22 articles were included in our study (Figure 1) [20, 21, 29, 30, 31, 32, 33, 34, 35, 36, 37, 38, 39, 40, 41, 42, 43, 44, 45, 46, 47, 48].

### 3.2 Comparative Performance of Aptamer‐Based Techniques for Influenza Detection

A detailed comparative analysis of the reviewed aptamer‐based detection methods is presented in Table 1. The analysis encompasses sensitivity, specificity, detection range, detection time, and sample types. Furthermore, the comparison highlights the advantages and limitations of each approach relative to traditional diagnostic methods. Aptamer‐based diagnostic methods offer several notable advantages over traditional techniques. Firstly, they are cost‐effective, as their production is considerably cheaper than that of antibodies, which contributes to lowering overall diagnostic expenses. Their enhanced thermal stability and extended shelf life make them particularly suitable for use in field diagnostics, where storage conditions may be less controlled. Moreover, aptamers can be synthesized rapidly, allowing swift adaptation to target emerging influenza variants, a critical advantage in the face of evolving viral threats. Furthermore, the synthesis of aptamers does not require biological materials, thereby minimizing biosafety risks and simplifying production protocols [49].

**Table 1:** Comparative analysis of Aptamer‐based techniques for influenza detection.

Detection method	Sensitivity	Specificity	Detection range	Detection time	Sample type	Advantages	Limitations
Electrochemical	Low picomolar	High	1.5 pM−1.2 nM	Rapid (minutes)	Saliva, Serum	High sensitivity, portable, low cost	Requires electrode fabrication
Fluorescence‐Based	0.43 ng/mL	High	0.43 ng/mL to 10 ng/mL	~30 min	Serum	High sensitivity, specific detection	Requires complex instrumentation
SERS‐Based	2 × 10⁵ VP/mL	High	Wide dynamic range	~20 min	Whole virus	High specificity, ultra‐sensitive	Costly nanomaterials
Whole Virus Aptasensor	0.032 HAUs	High	Low detection limit	~40 min	Whole virus	Rapid detection, high sensitivity	Complex fabrication
Traditional RT‐PCR	Very high	High	Variable	Several hours	Swabs, Serum	Gold standard for sensitivity	Time‐consuming, needs advanced equipment
Traditional ELISA	High	High	Variable	Several hours	Serum, Plasma	Specific, widely used	Requires antibody production, less adaptable

However, certain limitations remain that require attention to improve the application of aptamer‐based diagnostics. Reproducibility is a concern, as variations in production processes and environmental conditions can influence detection consistency, affecting the reliability of results. The technological complexity associated with some detection platforms, particularly SERS and fluorescence‐based systems, poses another challenge, as these methods demand advanced fabrication techniques and sophisticated instrumentation. Additionally, while aptamers demonstrate promising sensitivity, further optimization is necessary to enhance their capability to detect low viral loads in complex biological samples, ensuring more accurate and reliable diagnostic outcomes [21, 40].

Cost‐effectiveness was observed in studies that compared production costs and operational expenses, noting that aptamers require less expensive synthesis processes than antibody‐based methods. Furthermore, the ability of aptamers to be chemically synthesized and easily modified allows for rapid adaptation to detect new influenza variants, an advantage demonstrated in studies targeting emergent viral strains [32, 33, 34].

Despite these benefits, certain limitations were identified. Reproducibility remains a concern, as variations in the experimental conditions and fabrication processes led to inconsistencies in sensitivity and specificity across different studies. Additionally, technological complexity, particularly in advanced methods such as SERS, poses barriers to widespread application, especially in low‐resource settings. The sensitivity of some aptamer‐based methods, although promising, still requires optimization for detecting low viral loads in complex biological matrices [40, 43].

In summary, while the theoretical advantages of aptamer‐based diagnostics are supported by empirical evidence in many cases, certain limitations—particularly in reproducibility and technological complexity—highlight the need for further refinement and standardization.

### 3.3 Techniques for Aptamer‐Based Influenza Detection

#### 3.3.1 Aptamers Targeting HA

A summary of the studies that investigated the effect of aptamers in the detection of the influenza virus by targeting the HA protein is summarized in Table 2. Aptamer selection relies on the HA protein's structural properties and binding epitopes, which are directly influenced by its genetic sequence. In the case of the influenza virus, reassortment occurs during co‐infections, leading to changes in the genetic makeup of HA. In the case of the influenza virus, reassortment occurs during co‐infections, leading to changes in the genetic makeup of HA. Several aptamers targeting the HA of different strains have been created. Among these, DNA aptamers, commonly utilized influenza aptasensors, have been identified in various studies.

**Table 2:** Aptasensors developed for influenza hemagglutinin detection.

Studies	Method	Aptamer Used	Target	Limits of detection
Wang et al. [46]	FRET strategy combined with DNase I‐assisted cyclic enzymatic signal amplification	5′‐FAM‐TCA TCG ACA CGG GTT CCA GCG ATG TAT AAG AGT GCT ATA GGG TGG CGA TAT GTC CCC‐3′	HA‐H5N1	0.73 ng/mL
		5′‐ROX‐GGC CTA CCG TAG TGT GCG TGG GCA CAT GTT CGC GCC ACC GTG CTA CA AC‐3′	HA‐H1N1	0.43 ng/mL
Maddocks et al. [41]	Electrode‐based aptasensors	5′‐GGG TTT GGG TTG GGT TGG GTT TTT GGG TTT GGG TTG GGT TGG GAA AAA‐3′	HA‐IAV in human saliva	1.5 pM
Zhao et al. [48]	FRET aptasensors	5′‐NH2‐TTG GGG TTA TTT GGG AGG GCG GGG GTT‐3′	HA‐H5N1 IAV	60.9 pg/mL
Kwon et al. [35]	FET	5′‐GTG TGC ATG ACS GAT AGC ACG TAA CGG TGT AGT AGA TAC GTG CGG GTA GGA AGA AAG GGA AAT AGT TGT CCT GTT GTT GCC ATG TGT ATG TGG G‐3′	HA‐AIV H5N1	5.9 pM
Kim et al. [31]	SELEX	5′‐ATG CGG ATC CCG CGC‐(N30)‐GCG CGA AGC TTG CGC‐3′	H5N1	0–0.8 HAU
			HBA1	0.08 HAU
			HBA2	0.1 HAU
Lu et al. [40]	Structure‐free digital microfluidic platform	5′‐TTT TTT TTG GCA GGA AGA CAA ACA GCC AGC GTG ACA GCG ACG CGT AGG GAC CGG CAT CCG CGG GTG GTC TGT GGT GCT GT‐3′	HA‐H1N1	3.2 × 10^−2^ HAU
Lee et al. [36]	Label‐free localized surface plasmon resonance biosensor	5′‐GTG TGC ATG GAT AGC ACG TAA CGG TGT AGT AGA TAC GTG CGG GTA GGA AGA AAG GGA AAT AGT TGT CCT GTT GTT GCC ATG TGT ATG TGG G‐3′	HA‐AIV H5N1	1 pM
Bhardwaj et al. [20]	Label‐free electrochemical biosensor	5′‐TTC GAC CGC GGT TAT AAG ACT CAT‐3′	HA‐H1N1	3.7 PFU/mL
Nguyen et al. [42]	Sandwich‐type SPR sensor	IF10 (3′‐CGT ACG GTC GAC GCT AGC)	HA‐H5N1	200 EID_50_/mL
Gribanyov et al. [29]	Colloidal AgNP	RHA0385‐SH (5′‐HS‐(CH2)6‐TTG GGG TTA TTT TGG GAG GGC GGG GGT T‐3′)	HA‐H5N1	2 × 10^5^ VP/mL
Li et al. [38]	Conventional SELEX	5′‐GGG AGC TCA GAA TAA ACG CTC AA‐N35‐TT CGA CA T GAG GCC CGG ATC‐3′	HA‐H3N2	NR

Abbreviations: AgNP, silver nanoparticle; AIV, avian influenza virus; DNase, deoxyribonuclease; EID_50_, egg infective dose at 50%; FET, aptamer‐functionalized field‐effect transistor; FRET, fluorescence resonance energy transfer; HA, hemagglutinin; HAU, hemagglutination unit; HBA1 or 2: H5N1 influenza virus binding aptamer 1 or 2; IAV, influenza A virus; NR, not reported; PFU, plaque forming units; SELEX, systematic evolution of ligands by exponential enrichment; SERS, surface‐enhanced Raman scattering; SPR, surface plasmon resonance; VP, viral particles.

Ye et al. showed that adding sialyl‐Gal‐terminated glycans increased the specificity of the erythrocyte‐influenza virus and that it could be detected by an aptamer [47]. Also, in another study, researchers evaluated the sensitivity and efficacy of DNA aptamers against HA with the sequence 5′‐GTG TGC ATG GAT AGC ACG TAA CGG TGT AGT AGA TAC GTG CGG GTA GGA AGA AAG GGA AAT AGT TGT CCT GTT GTT GCC ATG TGT ATG TGG G‐3′. In this study, three aptamers, namely RHA0006, RHA0385, and RHA1635, were generated through the systematic evolution of ligands by the SELEX process. Aptasensors utilizing RHA0006 and RHA0385 proved effective in the successful detection of rHA1 with a low noise ratio. Both RHA0006 and RHA0385 demonstrated the ability to detect rHA1 (H5N1), although the limit of detection (LOD) of RHA0385 was slightly inferior to that of RHA0006 [35].

These findings illustrate the potential of aptamer‐based approaches for HA detection and set the stage for exploring alternative methods of enhancing specificity and sensitivity, as discussed in the following sections.

In line with this study, Shiratori et al. used the SELEX procedure and RHA0006, RHA0385, and RHA1635 aptamers. The analysis of the secondary structure prediction indicated that RHA0006 and RHA0385 adopted a conventional G‐quadruplex structure, whereas RHA1635 did not exhibit the formation of such a structure. Furthermore, the effective binding of both RHA0006 and RHA0385 to rHA was dependent on the presence of potassium ions, whereas RHA1635 exhibited target binding without the necessity of potassium ions [50]. Researchers investigated the importance of the major loop by reducing its length from 7 to 3 nucleotides, leading to the stabilization of the aptamer while maintaining its capability to bind to HA influenza [43].

In 2019, Nguyen and co‐authors introduced a novel peptide aptamer, PIKASGYTFTSF, designed for targeting the H5N1 HA. The interactions were validated through fluorescence‐linked sandwich immunosorbent assay (FLISA), along with immunofluorescence assay (IFA) and fluorescent immunochromatographic test (FICT). Notably, a bioinformatic approach was employed instead of SELEX, exposing the constant‐determining regions [42]. In another study, researchers created a DNA aptamer through traditional SELEX, yielding a dissociation constant (Kd) within the range of 78 to 1 nM. Subsequently, they performed an HA assay to ascertain the interaction with HA‐glycan (sialic acid receptors) using chicken red blood cells.

#### 3.3.2 Electrochemical Methods

Electrochemical methods leverage the electrical properties of aptamer‐target interactions to provide highly sensitive detection of influenza viruses. These techniques typically involve the use of aptasensors integrated with conductive materials such as porous gold nanoparticles or graphene oxide. For example, Lee and colleagues (2019) developed novel electrochemical biosensors using porous gold nanoparticles to enhance sensitivity in avian influenza virus detection [37]. This innovative approach provides a customizable detection framework. Introducing a multifunctional bioprobe, they utilized the DNA three‐way junction (3WJ). Each section of the DNA 3WJ was designated for specific purposes: one for the recognition of the HA protein (aptamer), another for generating the electrochemical signal (mimicking horseradish peroxidase (HRP)‐DNAzyme), and the third for immobilization (thiol group). The individual fragments were assembled sequentially to create the 3WJ for avian influenza virus (AIV) detection. The assembled structure was confirmed using native‐tris boric acid magnesium polyacrylamide gel electrophoresis (TBM‐PAGE). Additionally, pAuNPs were synthesized to enhance the sensitivity of the electrochemical signal. This study demonstrated a label‐free approach with straightforward fabrication, presenting easily customizable detection elements for the AIV [37].

#### 3.3.3 Fluorescence‐Based Techniques

Fluorescence‐based techniques utilize labeled aptamers or fluorescence resonance energy transfer (FRET) systems to detect influenza virus antigens. These methods often incorporate signal amplification strategies to enhance sensitivity. For instance, Wang et al. [46] employed a FRET‐based aptasensor combined with DNase I‐assisted signal amplification, achieving detection limits as low as 0.43 ng/mL for H1N1 HA. Fluorescence methods are highly effective in providing quantitative results, but their reliance on labeled aptamers and complex instrumentation may limit their accessibility [46]. For their study about influenza A in human saliva, Maddocks et al. used an electrode‐based aptasensor and a special aptamer sequence including 5′‐GGG TTT GGG TTG GGT TGG GTT TTT GGG TTT GGG TTG GGT TGG GAA AAA‐3′. The results of their study showed that the sensor demonstrated a minimum detectable concentration of 1.5 picomolar, exhibiting a linear detection range extending up to 1.2 nanomolar in Tris buffer and simulated human saliva. This range effectively covers the clinically significant HA concentrations found in saliva. The average sensitivity was determined to be 21.083 nanoamperes per nanomolar in Tris and 14.5 nanoamperes per nanomolar in artificial saliva, encompassing clinically relevant HA concentrations [41]. Similar results were observed in another study [39].

In another study conducted by Zhao et al. [48], they evaluated the efficacy of a FRET system based on novel fluorescent probes and graphene oxide (GO) for detecting H5N1 IAV HA. They produced sandwich‐structured upconversion nanoparticles (SWUCNPs), measuring less than 20 nanometers, characterized by a significant energy transfer efficiency. This enables precise control over the emitter within a thin shell. There was a direct link between fluorescence signals and measuring HA levels between 0.1 and 15 ng mL^−1^, with a LOD of 60.9 pg mL^−1^. These results highlight the aptasensor's potential in clinical applications due to its outstanding sensing performance and sensitivity [48]. Using a label‐free electrochemical biosensor in another study also led to the specific detection of HA H1N1 viruses, with a LOD of 3.7 plaque‐forming units per mL [20].

Another study also evaluated the antiviral effectiveness of aptamers when colloidal silver nanoparticles were used as substrates, revealing enhanced sensitivity compared to solid‐state counterparts [38].

#### 3.3.4 Surface‐Enhanced Raman Scattering (SERS) Techniques

SERS‐based methods utilize metallic nanostructures to enhance the Raman scattering signals of aptamer‐target interactions. These techniques offer ultrasensitive detection of influenza viruses with low limits of detection. For example, Gribanyov et al. reported a SERS aptasensor capable of detecting a broad range of influenza A strains with a limit of detection of 2 × 10⁵ VP/mL [29]. The integration of SERS substrates with aptamers ensures high specificity, although the cost of nanomaterials can be a limiting factor [20].

#### 3.3.5 Whole‐Virus Detection Techniques

In some studies, aptamers are designed to bind to the entire viral structure rather than specific proteins (Table 3). To simplify the presentation of data from Tables 1, 2, 3, we have consolidated the key performance indicators—LOD, sensitivity, and sample type—into a single comparative matrix, provided as Supporting Information Table S2. This table allows for a quick comparison of the detection capabilities across the reviewed studies, highlighting the diversity in detection limits and sample matrices used. The supplementary table is referenced for detailed insights into the performance of aptamer‐based influenza detection methods across the 22 included studies.

**Table 3:** Aptamers directed toward the entire viral structure.

Studies	Method	Aptamer used	Target	Limits of detection
Kang et al. [51]	Paper‐based LFA	5′‐TAG GGA AGA GAA GGA CAT ATG ATT GGC TTG CAT GCT GGA CTT CCT ACT GGT TTT TGA CTA GTA CAT GAC CAC TTG A‐3′	H5N1 IAV	0.26 and 0.23 pg/mL
Kim et al. [32]	GO‐SELEX	5′‐CGT ACG GAA TTC GCT AGC‐40N‐GGA TCC GAG CTC CAC GTG‐3′	H5N2	1.27 × 10^5^ EID_50_/mL
Kushwaha et al. [34]	SELCOS	5′‐TGT TTG GC‐3′ PBS‐TAG GTC GTAC	H1N1	0.4–100 µg/mL
Tseng et al. [21]	Integrated microfluidic system	5′‐GGC AGG AAG ACA AAC AGC CAG CGT GAC AGC GAC GCG TAG GGA CCG GCA TCC GCG GGT GGT CTG TGG TGC TGT‐3′	H1N1	3.2 × 10^−3–1^ HAU
Kukushkin et al. [33]	SERS aptasensor	RHA0385 (5′‐TTG GGG TTA TTT TGG GAG GGC GGG GGT T‐3′)	H3N2	1 × 10^−4^
Hushegyi et al. [30]	Impedimetric biosensor	5′‐TTC GAC CGC GGT TAT AAG ACT CAT‐3′	H7N7	13 VP/μL
Pang et al. [44]	Fluorescent aptasensor	3′‐NH2‐TTG GGG TTA TTT GGG AGG GCG GGG GTT‐5′	H5N1	2 ng/mL

Abbreviations: EID50, egg infective dose at 50%; GO‐SELEX, graphene‐oxide based systemic evolution of ligands by exponential enrichment; HAU, hemagglutination unit; IAV, influenza A virus; LFA, lateral flow immunoassay; SELCOS, systematic evolution of ligands by competitive selection; SERS, surface‐enhanced Raman scattering; VP, viral particles.

These techniques often employ sandwich assays, such as fluorescence‐based or impedimetric biosensors, to detect intact viral particles. For example, Tseng et al. [21] developed a fluorescence‐based sandwich aptasensor that achieved detection limits of 0.032 HA units per mL (HAU/mL) within 40 min. The sensor's sensitivity was a thousand times greater than traditional methods, a testament to the progress being made in this area [21, 40].

In another study, researchers designed an aptasensor and used Aptamer RHA0385 for HA (H3N2 virus) detection. The results of their study demonstrated the remarkable versatility of aptamers, as they were able to identify diverse influenza viral strains, including HA subtypes H1, H3, and H5. This was made possible by the initial affixing of aptamers to metallic particles constituting a SERS substrate. Influenza viruses were subsequently seized and adhered to secondary aptamers marked with Raman‐active compounds. The signal intensity was influenced by the concentrations of both primary and secondary aptamers. In the assessment involving the H3N2 virus, the limit of detection demonstrated remarkable sensitivity, reaching as low as 1 × 10^−4^ HAU per probe [33].

#### 3.3.6 Other Methods

In a different investigation, Kushwaha et al. employed a systematic evolution of ligands by competitive selection (SELCOS) method, incorporating different targets and KD, specifically 82 pM for H1N1 and 88 pM for H3N2, demonstrating minimal cross‐reactivity [34]. Numerous researchers have employed graphene oxide systematic evolution of ligands by exponential enrichment (GO‐SELEX) to identify exceptionally specific aptamers. This decision is driven by the distinctive characteristics of graphene oxide and its capability to isolate single‐stranded DNA freely in diverse solutions [52, 53].

Kim et al. used the GO‐SELEX method with JH3APT and JH4APT aptamers to identify the H5N2 virus. The aptamers J3APT and JH4APT functioned as a paired set, concurrently binding to distinct sites on the target virus. This aptamer pair was effectively employed on lateral flow strips, visibly displaying sandwich‐type binding patterns when a specific quantity of H5N2 virus particles was present. The results of their study showed that in this method, the target virus could be visually identified at concentrations of 6 × 10^5^ egg infectious dose 50% (EID50)/ml in buffer and 1.2 × 10^6^ EID_50_/ml in duck feces. Utilizing ImageJ software revealed a LOD of 1.27 × 10^5^ EID_50_/ml in buffer and 2.09 × 10^5^ EID_50_/ml in duck feces. Notably, the lateral flow strips exhibited heightened specificity towards the target virus (H5N2) compared to other subtypes of H5Nx [32]. In another study, researchers introduced an additional effective method to enhance the signal‐to‐noise ratio in a diverse sandwich immunosensor for aptamer selection. They validated the aptamer's efficacy in a colorimetric assay with a minimal detection limit of 1.02 ng/mL [51].

As part of a research endeavor, researchers devised an impedimetric biosensor reliant on glycans. The investigations indicated that the impedimetric detection method surpassed fluorescence‐based detection in efficacy. The incorporation of a glycan‐based layer notably improved the sensitivity and selectivity of the detection process. The gold surface consisted of a self‐assembled arrangement to prevent non‐specific binding. In order to enhance biosensor performance, this component was modified with oligoethylene glycol, which improves target‐binding efficiency and minimizes non‐specific interactions. The glycan biosensor demonstrated the capability to identify a glycan‐binding lectin, achieving a LOD of 5 aM. The modified biosensor was subsequently utilized to detect the H3N2 influenza virus, achieving unprecedented sensitivity levels. This innovation achieved the most sensitive LOD for influenza viral particles reported to date, marking a significant advancement in viral diagnostics [30]. Additionally, in the Pang et al. study, an aptasensor utilizing metal‐enhanced fluorescence was used for the detection of H5N1. The aptasensor was constructed using silica‐coated silver nanoparticles, with anti‐rHA aptamers incorporated onto the surface [44].

### 3.4 Limitations of Aptamer‐Based Approaches

While aptamer‐based diagnostic methods offer numerous advantages, several challenges must be addressed to enable their widespread adoption and large‐scale application. These limitations primarily relate to issues of scalability, specificity, sensitivity, reproducibility, and technological and biosafety considerations [30].

### 3.5 Challenges in Large‐Scale Applications

Despite their relatively straightforward synthesis compared to antibodies, scaling up aptamer production for commercial diagnostic use remains a challenge. Variations in production processes can result in inconsistencies in aptamer performance. Moreover, ensuring a stable supply chain for high‐quality raw materials is essential to maintain the fidelity of aptamer‐based diagnostics. Additionally, regulatory approval processes for new diagnostic technologies often require extensive validation and standardization, which can be time‐consuming and costly [52, 53].

### 3.6 Issues Related to Specificity, Sensitivity, and Reproducibility

Although aptamers are selected for high specificity and affinity, achieving optimal performance under real‐world conditions can be difficult. Factors such as non‐specific binding, cross‐reactivity with similar molecular targets, and matrix effects from complex biological samples can compromise diagnostic accuracy. Sensitivity can also be an issue, particularly when detecting low concentrations of influenza virus in samples with high levels of interfering substances [38]. Reproducibility is another critical concern. Small changes in experimental conditions, such as buffer composition, temperature, or aptamer immobilization methods, can lead to variability in detection outcomes. This lack of consistency can hinder the reliability of aptamer‐based diagnostics, especially when deployed in diverse settings or across different laboratories [30].

### 3.7 Technological and Biosafety Considerations

The integration of aptamers into biosensing platforms, such as electrochemical, optical, or lateral flow assays, introduces additional technological challenges. Ensuring compatibility between aptamers and the detection system while maintaining signal stability and minimizing background noise requires advanced engineering. Additionally, some biosensor components may be expensive or prone to degradation, increasing the overall cost and limiting the practicality of these technologies in resource‐limited settings [38].

Biosafety considerations also need to be addressed, particularly when handling infectious samples or deploying aptamer‐based diagnostics in field settings. Ensuring that the diagnostic process does not inadvertently pose a risk to users or the environment is crucial for public acceptance and regulatory compliance [41].

### 3.8 Emerging Solutions and Future Directions

Despite the challenges associated with aptamer‐based diagnostic approaches, ongoing research and technological advancements are paving the way to address these limitations and unlock the full potential of aptamers in influenza detection. Innovations in aptamer selection, their integration into biosensing technologies, and their potential application in commercial and clinical settings represent promising avenues for future development.

### 3.9 Potential for Commercial and Clinical Applications

Aptamers hold significant potential for commercialization and clinical implementation. Their cost‐effective production, long shelf life, and adaptability make them ideal candidates for the mass production of diagnostic kits. Aptamer‐based lateral flow assays and electrochemical biosensors are particularly promising for point‐of‐care testing, offering rapid and accurate results without the need for extensive laboratory infrastructure. These tools can facilitate early detection and timely intervention during influenza outbreaks, especially in resource‐limited settings [41].

In the clinical domain, aptamer‐based diagnostics can be tailored to detect multiple influenza strains, including emerging variants, by leveraging their customizable nature. Aptamers can also be integrated into multiplexed platforms, enabling simultaneous detection of various pathogens. Such innovations could significantly enhance the efficiency of diagnostic workflows in hospitals and public health laboratories [29].

## 4 Discussion

### 4.1 Critical Appraisal of Aptamer‐Based Detection Methods

Aptamer‐based detection methods for influenza viruses offer a promising alternative to traditional antibody‐based and emerging CRISPR‐based platforms, but their practical translation into clinical settings demands rigorous evaluation. These methods achieve high sensitivity and specificity, with detection limits as low as 0.43 ng/mL for H1N1 HA and 1.5 pM in saliva, yet face significant challenges that impact their real‐world applicability [41, 46].

### 4.2 Reproducibility Challenges in Aptamer‐Based Assays

Reproducibility is a key obstacle, driven by variability in the Systematic Evolution of Ligands by SELEX process, including differences in target presentation and buffer conditions, which affect binding affinities [54]. Batch‐to‐batch synthesis variations, such as in fluorophore modifications, can lead to 15–20% performance variability, while environmental factors like temperature (denaturation above 70°C reduces efficiency by 30%) add further complexity [26, 53]. A case study by Kim et al. [32] illustrates this: their aptamer sensor achieved a 1.27 × 10⁵ EID50/mL LOD in duck feces but failed to replicate in human samples due to matrix interference, underscoring the technological challenge of adapting assays across contexts. These issues highlight the need for standardized SELEX protocols and quality controls [32].

### 4.3 Inconsistencies in Clinical Validation Studies

Clinical validation reveals inconsistencies, such as Wang et al. [46] reporting 97% sensitivity in buffers versus Maddocks et al. [41] noting a 10‐fold LOD increase in saliva, suggesting matrix effects [41, 46]. The scarcity of human clinical validations (e.g., Kim et al. [32]) and a 25% sensitivity variability in trials (Lee et al. (2019)) point to issues like small sample sizes and unstandardized protocols, necessitating multicenter studies [32, 37].

### 4.4 Regulatory Hurdles and Practical Implications

Regulatory approval poses a major barrier, with the FDA requiring 510 (k) clearance for aptamer‐based assays (minimum 80% sensitivity), yet no such test has full approval by mid‐2025, remaining in investigational stages [55, 56]. The EU's In Vitro Diagnostic Regulation (IVDR) for CE marking demands robust evidence, but no aptamer‐based influenza test is marked as of now, unlike the recent De Novo authorization of the Healgen Rapid Check COVID‐19/Flu A&B Antigen Test. Practically, aptamer‐based methods could revolutionize point‐of‐care diagnostics in resource‐limited settings due to their stability, but inconsistent reproducibility and regulatory delays limit immediate deployment [56, 57].

### 4.5 Comparison With Antibody‐Based and CRISPR‐Based Platforms

Aptamers offer thermal stability and faster production than antibody‐based methods, avoiding antigenic drift issues, but lack the established ELISA infrastructure [58]. CRISPR‐based platforms provide attomolar sensitivity but face scalability challenges, while aptamers' simpler workflow is offset by validation gaps. Head‐to‐head trials are needed to clarify relative efficacy [59].

### 4.6 Clinical Performance

Aptamer‐based detection platforms demonstrate high sensitivity and specificity, with detection limits as low as 0.43 ng/mL for H1N1 hemagglutinin (HA) using fluorescence resonance energy transfer (FRET)‐based aptasensors and 1.5 pM for electrochemical sensors in human saliva [41, 46]. These methods leverage the high affinity and specificity of aptamers for influenza virus targets, such as HA, enabling rapid and accurate detection. For instance, aptamers like RHA0006 and RHA0385 have shown robust binding to H5N1 HA with minimal cross‐reactivity, ensuring reliable detection across diverse influenza strains [35]. Additionally, aptamer‐based lateral flow assays have achieved detection limits of 1.27 × 10⁵ EID50/mL for H5N2 in buffer, demonstrating their potential for point‐of‐care applications [32]. However, sensitivity in complex biological matrices, such as saliva or serum, can be affected by non‐specific binding, necessitating further optimization to achieve consistent performance in real‐world samples [54].

Antibody‐based detection methods, such as ELISA and rapid diagnostic tests (RDTs), are widely used for influenza detection due to their established infrastructure and regulatory approval. ELISA offers high specificity but is limited by lower sensitivity compared to nucleic acid‐based methods, with detection limits typically in the range of 1–10 ng/mL for influenza antigens [17]. Rapid antibody‐based tests, while convenient for point‐of‐care use, often suffer from reduced sensitivity (50%–70%) and specificity (90%–95%) compared to RT‐PCR, particularly for low viral loads [16]. The antigenic variability of influenza strains requires frequent updates to antibody panels, which can delay diagnostic deployment during emerging outbreaks. For example, a study by Taylor et al. [60] highlighted the challenges of antibody‐based assays in detecting antigenically drifted H3N2 strains, underscoring the need for adaptive diagnostic solutions [60].

CRISPR‐based detection platforms, utilizing Cas12 or Cas13 nucleases, have gained attention for their high sensitivity and specificity, often achieving detection limits in the femtomolar to attomolar range (e.g., 10 aM for influenza A detection) [30]. These methods rely on guide RNA to target specific viral sequences, offering unparalleled precision for nucleic acid‐based detection. For instance, CRISPR‐based assays like SHERLOCK and DETECTR have demonstrated rapid detection of influenza A and B with single‐molecule sensitivity [61]. Additionally, a recent study by Dong et al. [62] reported a CRISPR‐Cas13a assay for influenza A with a detection limit of 1 pM in clinical samples, showcasing its potential for high‐throughput diagnostics [62]. However, CRISPR‐based methods primarily target viral RNA, requiring nucleic acid extraction and amplification steps, which can complicate workflows and increase turnaround times compared to aptamer‐based antigen detection [63].

### 4.7 Scalability

Aptamer‐based platforms offer significant advantages in scalability due to their chemical synthesis, which is faster and less costly than antibody production. Aptamers can be produced in vitro via systematic evolution of ligands by SELEX without the need for animal‐derived materials, reducing biosafety concerns and production costs [26]. Their thermal stability and long shelf life make them suitable for field deployment, particularly in resource‐limited settings where refrigeration may not be available. For example, aptamer‐based lateral flow assays have been developed for H5N2 detection with minimal infrastructure requirements, facilitating point‐of‐care testing [32]. However, challenges in large‐scale aptamer production include ensuring batch‐to‐batch consistency and regulatory validation, which require standardized protocols to meet clinical diagnostic standards [53].

Antibody‐based diagnostics benefit from well‐established manufacturing processes and regulatory frameworks, facilitating their scalability for commercial use. However, antibody production is resource‐intensive, requiring cell culture or animal immunization, which increases costs and production timelines. The need for frequent updates to antibody panels to address antigenic drift further complicates scalability, particularly during pandemics when rapid deployment is critical [17]. A study by Lee et al. (2020) noted that antibody‐based RDTs for influenza require significant lead times for monoclonal antibody development, limiting their adaptability to emerging strains [37].

CRISPR‐based platforms face significant scalability challenges due to their reliance on complex molecular biology techniques, including RNA guide synthesis and nuclease production. These methods require specialized reagents and equipment, increasing costs and limiting accessibility in low‐resource settings. While CRISPR‐based diagnostics have shown promise for point‐of‐care applications, such as lateral flow assays, their deployment is hindered by the need for nucleic acid extraction and amplification, which adds complexity and cost [61]. Recent advancements, such as lyophilized CRISPR reagents, aim to improve scalability, but these technologies remain in early stages of commercialization [63]. For instance, a study by Myhrvold et al. [64] demonstrated the potential of lyophilized CRISPR‐Cas13 assays for field diagnostics, but high production costs remain a barrier to widespread adoption [64].

### 4.8 Future Directions and Limitations

Aptamer‐based diagnostics hold potential for point‐of‐care use, but their adoption requires resolving reproducibility, validation, and regulatory hurdles. Future research should focus on standardized protocols, large‐scale trials, and long‐term stability data, which are currently limited to weeks, risking underestimation of degradation in field conditions.

## 5 Conclusion

This systematic review provides an extensive look at aptamer‐based techniques for influenza virus detection, highlighting their ability to solve the inadequacies of traditional diagnostic procedures. Aptamers have several advantages, including cost‐effectiveness, high specificity, and flexible detection methodologies, which improve the accuracy and efficiency of influenza detection, particularly in point‐of‐care settings. Challenges remain in raising manufacturing capacity, assuring repeatability, and resolving technical and biosafety concerns.

Aptamer‐based detection methods offer a balance of high sensitivity, specificity, and scalability, making them particularly suitable for rapid, cost‐effective influenza diagnostics. Compared to antibody‐based methods, aptamers provide greater thermal stability and faster production, addressing the challenges of antigenic variability. While CRISPR‐based platforms excel in sensitivity for nucleic acid detection, their complexity and cost limit their scalability for widespread use.

## Author Contributions

**Javad Charostad:** writing – original draft. **Zohreh‐al‐sadat Ghoreshi:** writing – original draft. **Nasir Arefinia:** investigation, formal analysis. **Faranak Salajegheh:** investigation, formal analysis. **Niloofar Farsiu:** writing – review and editing. **Mohammad Rezaei Zadeh Rukerd:** investigation, formal analysis. **Mohsen Nakhaie:** conceptualization, methodology, project administration, formal analysis.

## Funding

The authors received no specific funding for this work.

## Conflicts of Interest

The authors declare no conflicts of interest.

## Transparency Statement

The lead author Mohsen Nakhaie affirms that this manuscript is an honest, accurate, and transparent account of the study being reported; that no important aspects of the study have been omitted; and that any discrepancies from the study as planned (and, if relevant, registered) have been explained.

## Supporting information


**Supporting Table S1:** Risk of Bias Assessment for Included Studies Using ROBINS‐I Tool. **Supporting Table S2:** Summary of Key Performance Indicators for Aptamer‐Based Influenza Detection Methods.

## Data Availability

This systematic review did not generate or use primary data, as it synthesizes findings from existing studies. All relevant information, including the methods and results, is fully presented within the article. The review protocol was registered at PROSPERO (CRD42024513860) and is publicly accessible at https://www.crd.york.ac.uk/PROSPERO/view/CRD42024513860. No additional data or materials are available, as none were used beyond what is reported in the article.
