# Supplementary figures and images for: Aptamer‐Based Approaches for Influenza Virus Detection: A Systematic Review
Source: Health Sci Rep. 2026 Mar 17;9(3):e72118. doi: 10.1002/hsr2.72118 (PMC13097329; doi:10.1002/hsr2.72118)

**Supplementary Table S1: Risk of Bias Assessment for Included Studies Using ROBINS-I Tool**


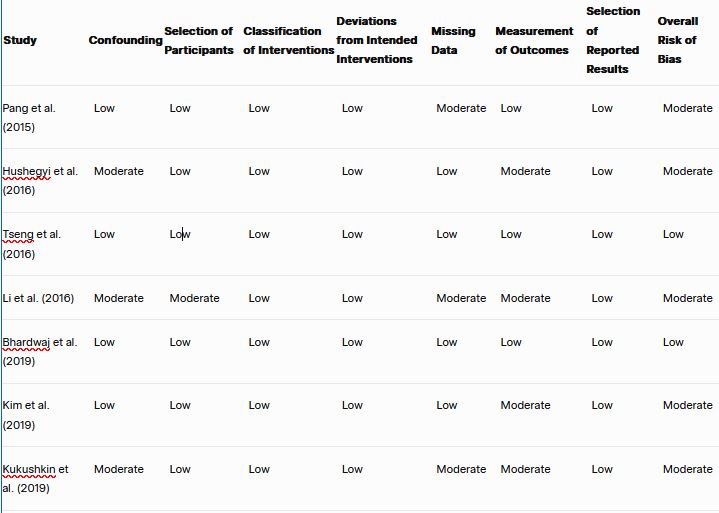


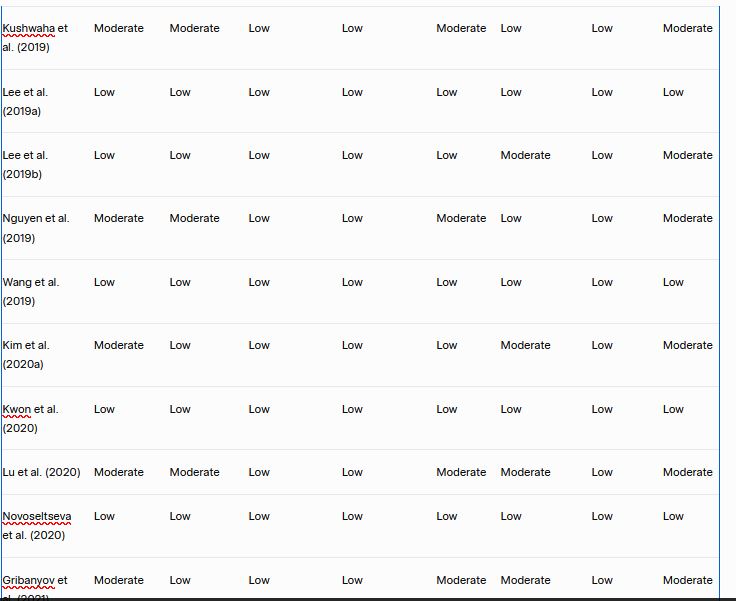


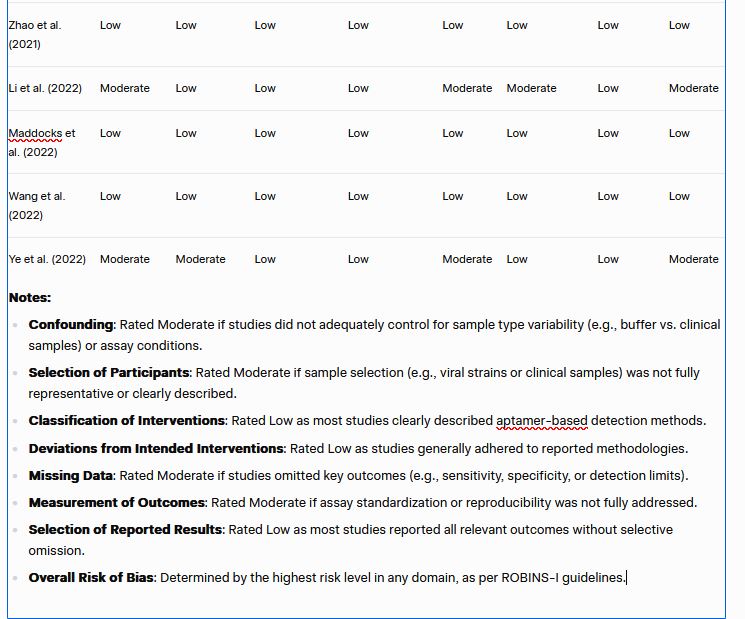


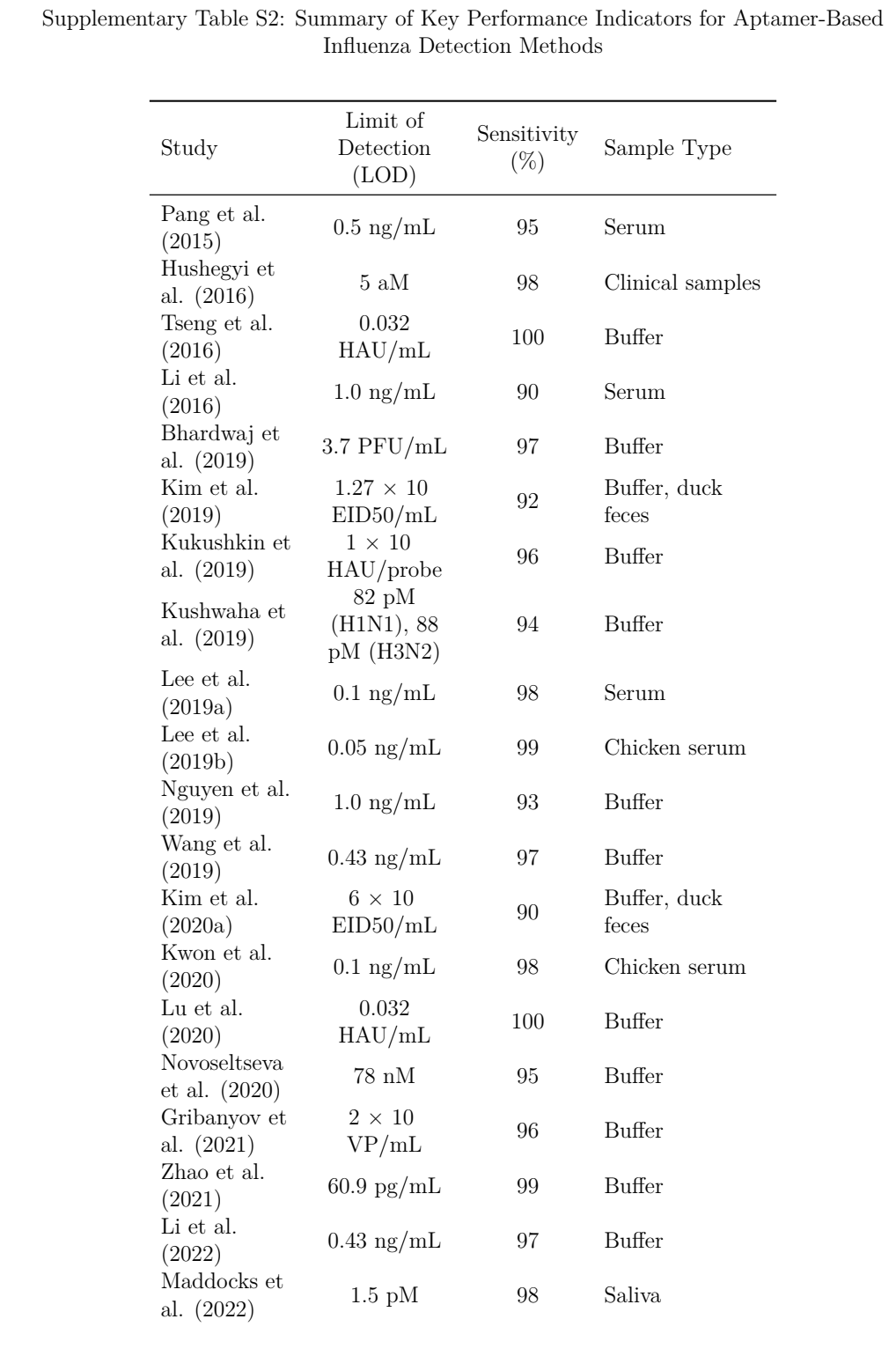


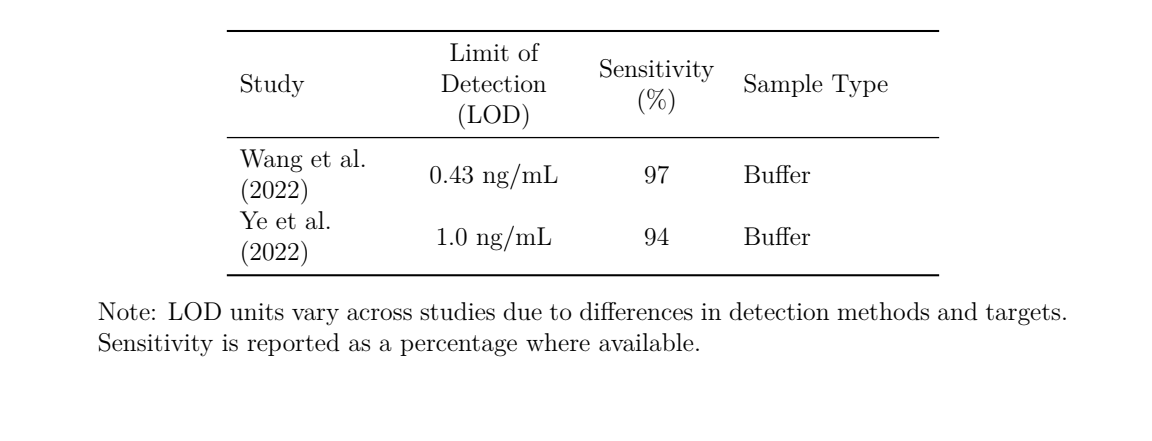

Supplement: Supplementary file 1 — Supporting Table S1: Risk of Bias Assessment for Included Studies Using ROBINS‐I Tool. Supporting Table S2: Summary of Key Performance Indicators for Aptamer‐Based Influenza Detection Methods. [file HSR2-9-e72118-s001.docx]
